# Bioinformatical analysis of the key differentially expressed genes and associations with immune cell infiltration in development of endometriosis

**DOI:** 10.1186/s12863-022-01036-y

**Published:** 2022-03-18

**Authors:** Shengnan Chen, Xiaoshan Chai, Xianqing Wu

**Affiliations:** Department of Obstetrics and Gynecology, The Second Xiangya Hospital of Central South University, Changsha, 410011 China

**Keywords:** Endometriosis, Gene expression omnibus, Bioinformatics, Immune cell infiltration

## Abstract

**Background:**

This study explored the key genes related to immune cell infiltration in endometriosis.

**Results:**

The Gene Expression Omnibus (GEO) datasets (GSE7305, GSE7307, and GSE11691), containing a total of 37 endometriosis and 42 normal tissues, were retrieved and analyzed to determine the differentially expressed genes (DEGs). Gene ontology (GO) annotations and Kyoto Encyclopedia of Genes (KEGG) analysis were performed to identify the pathways that were significantly enriched. The xCell software was used to analyze immune cell infiltration and correlation analyses were performed to uncover the relationship between key genes and immune cells. The analysis identified 1031 DEGs (581 upregulated and 450 downregulated DEGs), while GO analysis revealed altered extracellular matrix organization, collagen-containing extracellular matrix, and glycosaminoglycan binding and KEGG enrichment showed genes related to metabolic pathways, pathways in cancer, phosphatidylinositol 3-kinase-protein kinase B (PI3K-Akt) signaling, proteoglycans in cancer, and the mitogen-activated protein kinase (MAPK) signaling pathway. Furthermore, the protein–protein interaction network revealed 10 hub genes, i.e., *IL6*, *FN1*, *CDH1*, *CXCL8*, *IGF1*, *CDK1*, *PTPRC*, *CCNB1*, *MKI67*, and *ESR1*. The xCell analysis identified immune cells with significant changes in all three datasets, including CD4^+^ and CD8^+^ T cells, CD8^+^ Tem, eosinophils, monocytes, Th1 cells, memory B-cells, activated dendritic cells (aDCs), and plasmacytoid dendritic cells (pDCs). These 10 hub genes were significantly associated with at least three types of immune cells.

**Conclusions:**

Aberrant gene expression was related to abnormal infiltration of different immune cells in endometriosis and was associated with endometriosis development by affecting the tissue microenvironment and growth of ectopic endometrial cells.

## Background

Endometriosis is a benign gynecological condition characterized by the abnormal presence and growth of endometrial tissue outside the uterus. The disease most frequently occurs in the ovaries, fossa ovarica, uterosacral ligaments, and posterior cul-de-sac [1] or in rare cases, in the diaphragm, pleura, and pericardium [2]. Approximately 10% of childbearing-age women may be subject to endometriosis [3]. The main clinical symptoms of endometriosis include pelvic pain, dysmenorrhea, sexual difficulty, dysuria, and infertility [4]. However, to date, endometriosis pathogenesis remains to be defined, although the underlying molecular mechanism could be genetic, environmental, or immune-related [5]. Endometriosis was first discovered microscopically by Karl von Rokitansky in 1860 [5]. Sampson JA proposed the endometrial implantation theory (or the retrograde menstruation theory) for the development of endometriosis in 1927 [6], i.e., during menstruation, endometrial epithelium and stromal cells mixed in the menstrual blood could flow backward through the Fallopian tubes into the abdominal cavity and implant in the ovary and pelvic peritoneum, some of which could proliferate and spread to form endometriosis. Normally, the immune defense system in the peritoneum can suppress such a situation, like attachment and growth of refluxed cells. Indeed, although menstrual reflux occurs in more than 90% of women, only 6%-10% develop the disease [7]. Therefore, this theory alone may not fully explain endometriosis development, and other factors, including genetic, immunological, stem cell migration-related factors, could also play a role in endometriosis development [8–10].

To date, a great number of studies have shown that abnormal immunity could play an important role in endometriosis development; for example, the immune cells in the abdominal cavity are the first line of the body’s defense system against novel antigens entering the abdominal cavity. Changes in these immune cells, including monocytes, macrophages, natural killer (NK) cells, or other cytotoxic lymphocytes in the abdominal cavity, occur in endometriosis patients and the subsequent defense could be aberrant [11, 12], resulting in the transformation and growth of ectopic endometrial cells and endometriosis development. Moreover, these ectopic endometrial cells can release cytokines and inflammatory mediators and change the local peritoneum microenvironment to further promote endometriosis development. Since endometriosis development is a tissue-specific phenomenon, the local microenvironment obviously plays a role in endometriosis formation, in addition to the abdominal environment and body defense system, e.g., the ovary, which has high hormone levels, is an ideal site for a high frequency of endometriosis [13]. Secretion of immune-related cytokines and immune cell infiltration are also important to promote ectopic endometrial adhesion, angiogenesis, and matrix remodeling during endometriosis development [14–16]. In this regard, aberrant presence of immune cells, types, and functions was reported to be associated with endometriosis pathogenesis [17] and the affected cells included lymphocytes, macrophages, dendritic cells, NK cells, neutrophils, and eosinophils [18–20].

In this study, we utilized the online xCell tool to analyze the infiltration of 22 different immune cell subtypes between endometriosis and normal tissues [21]. After obtained the HUB gene associated with endometriosis with the R software, we then analyzed the association between HUB gene and immune cells with significant difference. Because endometriosis is a chronic inflammatory disease and lacks the effective diagnostic markers, we tried to provide the related genes for early and non-invasive diagnosis of endometriosis in future and for further study of the possible immune mechanism in endometriosis development.

## Results

### Identification of infiltrating immune cell subtypes in endometriosis

**Fig. 1:**
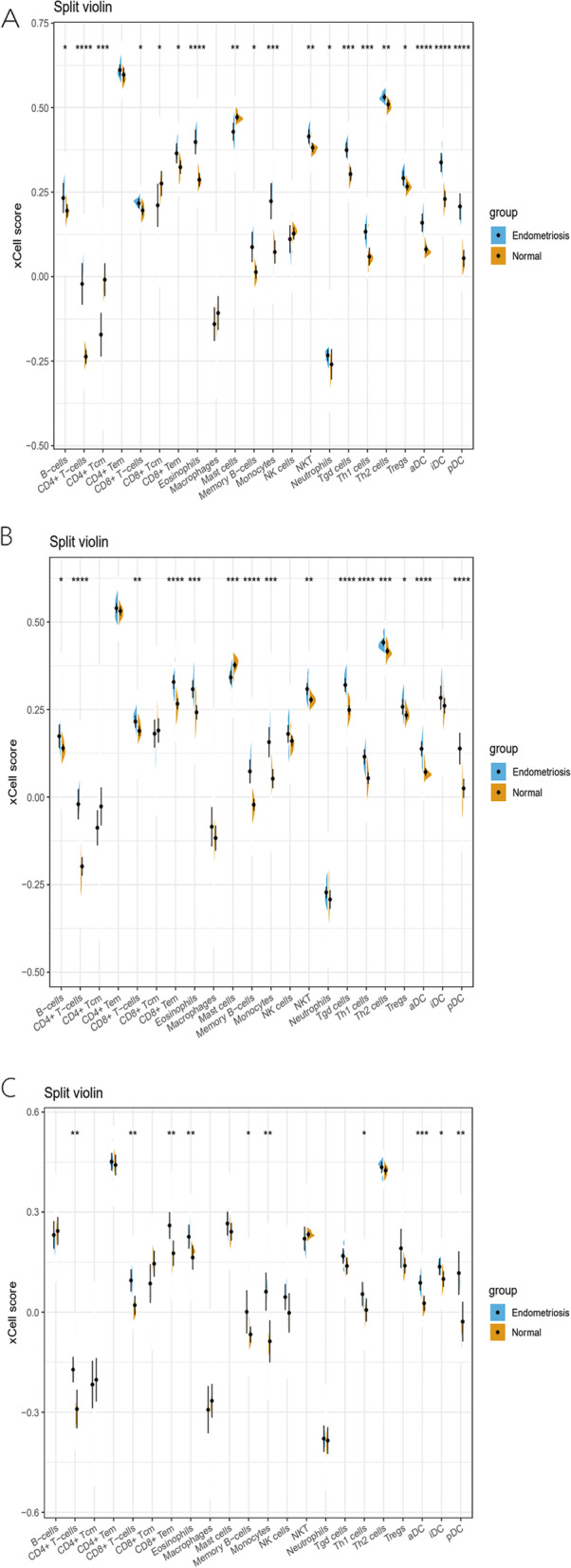
The xCell scores of 22 different subtypes of immune cells in endometriosis vs. normal tissues. **A** GSE7305 dataset; (**B**) GSE7307 dataset; (**C**) GSE11691 dataset

In this study, we included 37 cases of endometriosis and 42 cases of normal endometrium obtained from the GSE7305, GSE7307, and GSE11691 datasets. The diseased samples consisted of 28 cases of ovarian endometrioma and 9 cases of peritoneal endometriosis. All surgical samples were taken before any medications, such as hormone therapy. We first determined the cell types potentially involved in endometriosis in the three GEO datasets (GSE7305, GSE7307, and GSE11691) using the xCell tool analysis with the “Charoentong signatures (*N* = 22)” selected as the gene signatures [21]. We then plotted the split violin diagrams to visualize differences in immune cell infiltration using the cut-off value of *p* < 0.05 (Fig. 1). Our data showed nine significantly different immune cell types in the GSE7305, GSE7307, and GSE11691 datasets. The xCell scores for these nine different immune cell subtypes in endometriosis were significantly higher than those of the normal endometrium (Fig. 1).

### Profiling of differentially expressed genes in endometriosis

**Fig. 2:**
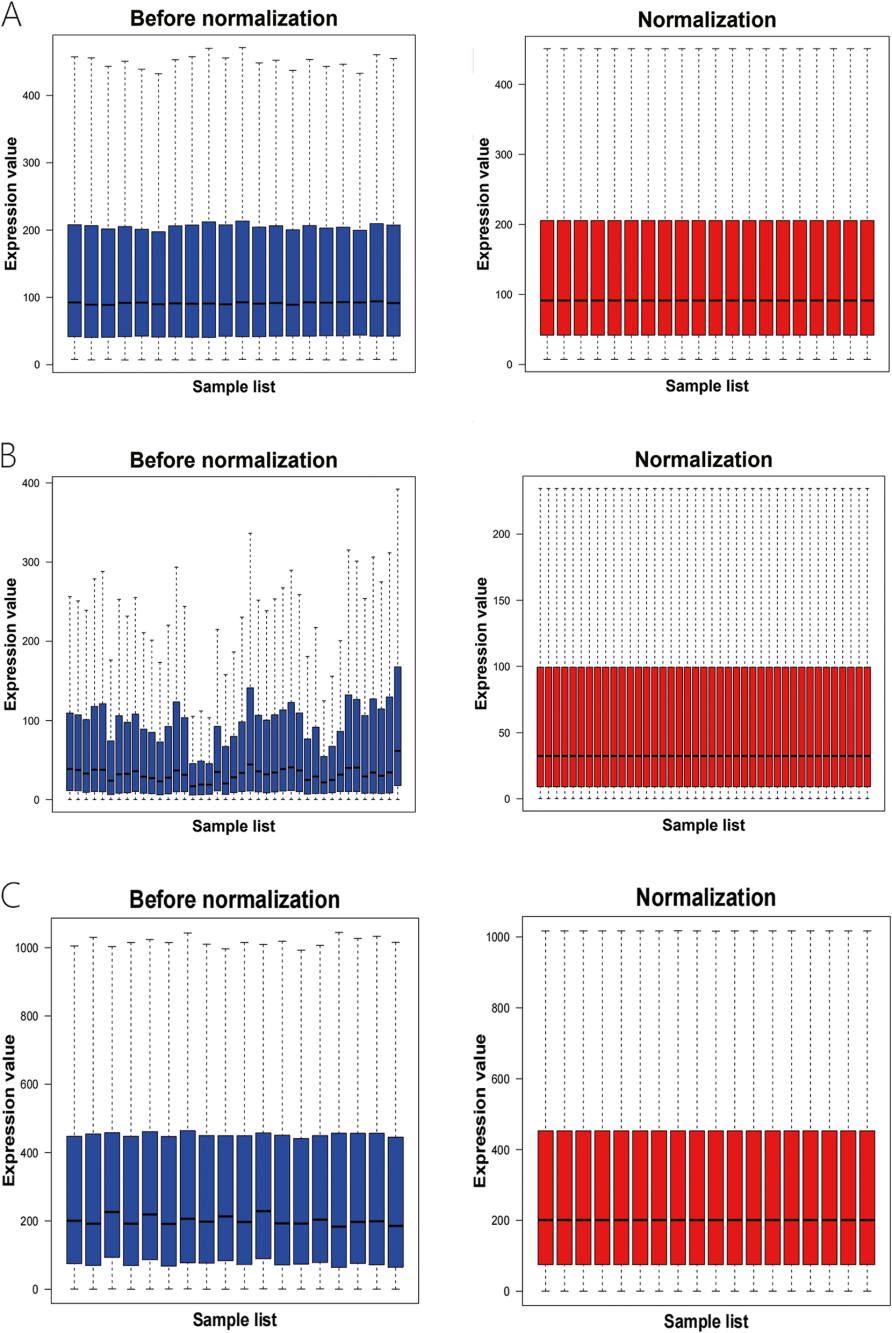
Profiling of DEGs in the GEO GSE7305, GSE7307, and GSE11691 datasets. **A** GSE7305; (**B**) GSE7307; (**C**) GSE11691 datasets. The blue bars represent the data before normalization, whereas the red bars show the data after normalization

**Fig. 3:**
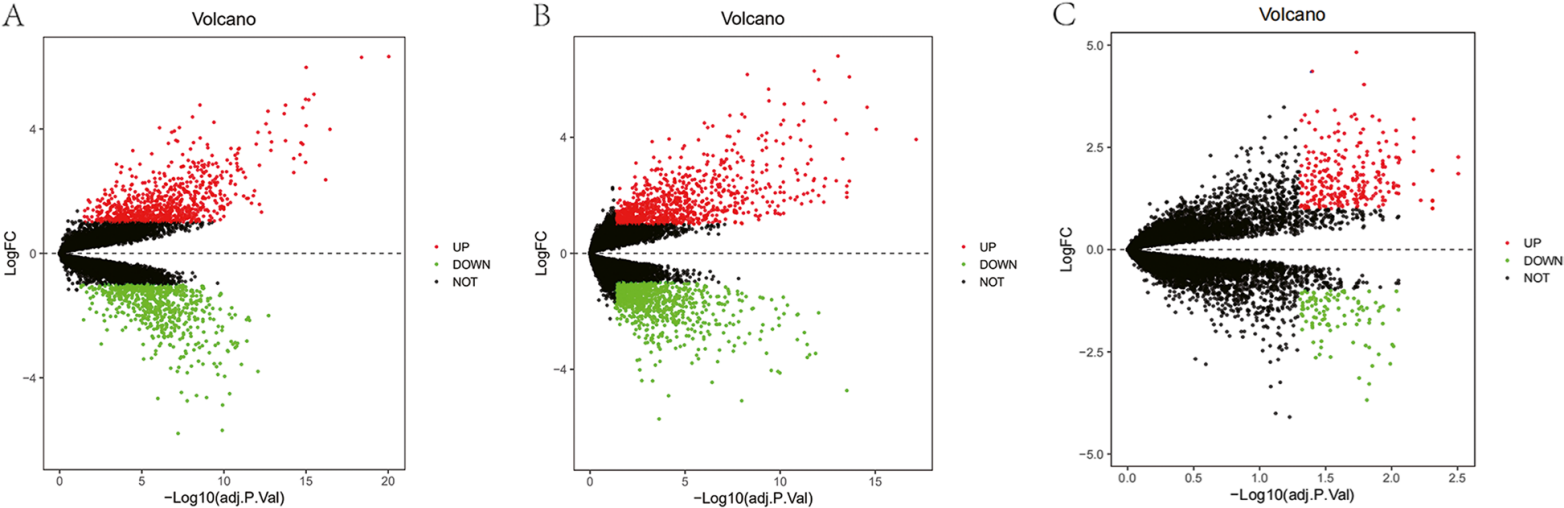
The volcano map of the DEGs in the GEO GSE7305, GSE7307, and GSE11691 datasets. **A** GSE7305; (**B**) GSE7307; (**C**) GSE11691 datasets. The red dots represent the upregulated DEGs using the cut-off values of adjusted *p* < 0.05 and |log fold change|> 1, whereas the green dots show the downregulated DEGs using the cut-off values of adjusted *p* < 0.05 and -|log fold change|< -1. The black spots represent genes with no significant difference in expression

**Fig. 4:**
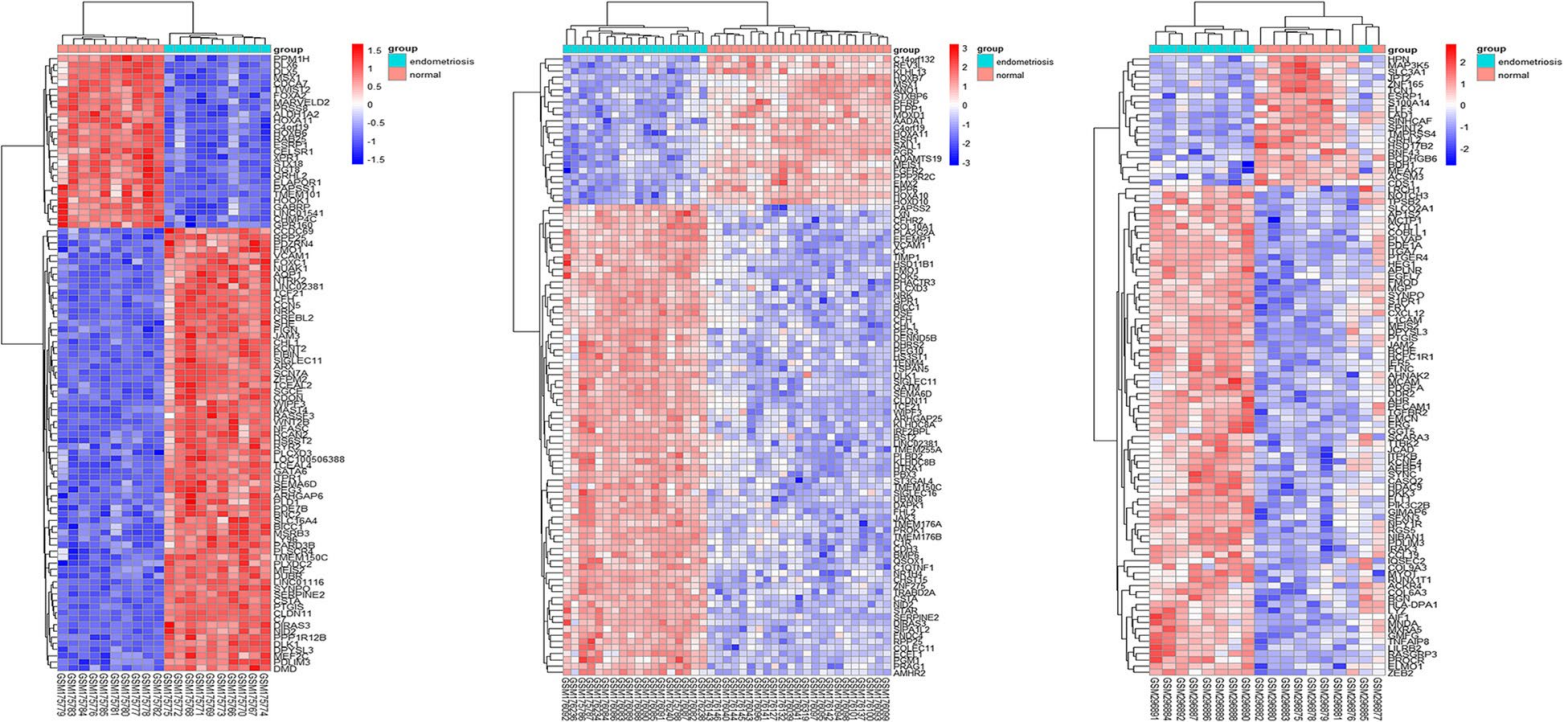
The cluster heatmaps of the top 100 DEGs in the GEO GSE7305, GSE7307, and GSE11691 datasets. **A** GSE7305, (**B**) GSE7307, and (**C**) GSE11691 datasets. The red color indicates relative upregulated DEGs, whereas the blue color shows the relative downregulated DEGs. The white color indicates no significant change in gene expression

After downloading the gene chip analytic data, we normalized the gene expression and the data are shown in Fig. 2. We then utilized the limma R package to screen and identify the DEGs using the criteria of adjusted *p* < 0.05 and |log fold change (FC)|> 1. The GSE7305 dataset contained 1,446 DEGs (813 upregulated and 633 downregulated DEGs), GSE7307 consisted of 1,782 DEGs (934 upregulated and 848 downregulated DEGs), and GSE11691 profiled a total of 367 DEGs (265 upregulated and 102 downregulated DEGs). The volcano map for the DEGs in these three dataset is shown in Fig. 3 and the cluster heat maps of the top 100 DEGs in each dataset are presented in Fig. 4.

**Fig. 5:**
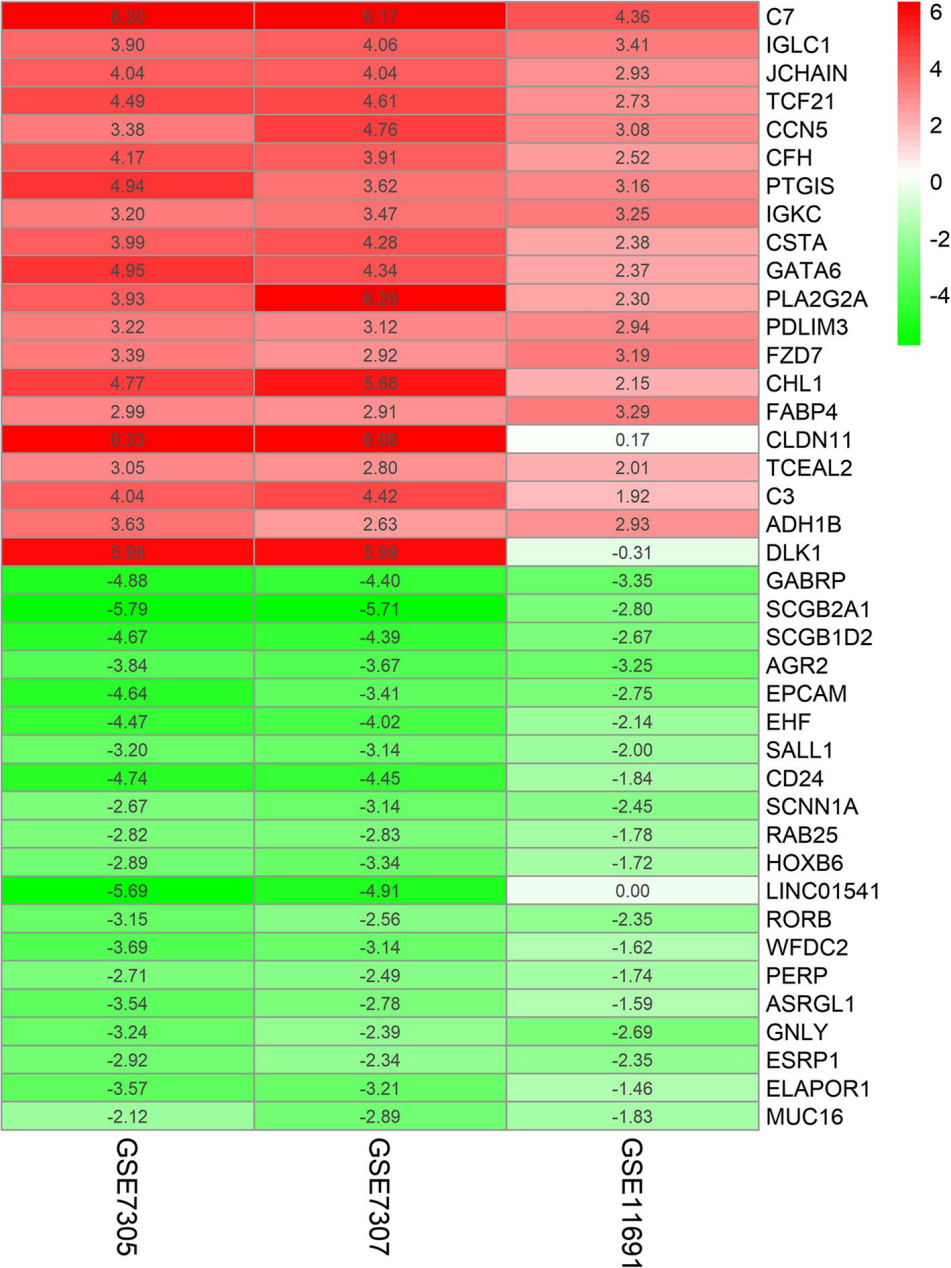
Heatmap of the top 20 upregulated and downregulated genes after RRA ranking analysis of all DEGs in the GEO GSE7305, GSE7307, and GSE11691 datasets. The red shaded text represents log FC > 0, while the green shaded text represents logFC < 0, and the value in the box represents the log FC value

We utilized the Robust Rank Aggregation method (RRA) according to a previous study [22] to analyze the DEGs in the GEO GSE7305, GSE7307, and GSE11691 datasets. RRA analysis theoretically assumes that each gene in each dataset is randomly arranged (expressed), but if a given gene ranks high in all datasets, the associated *p* value will be lower, indicating that the potential for the expression of this DEG is greater. After RRA ranking analysis with a corrected *p* < 0.05 and logFC > 1 or − logFC <  − 1, we identified 1031 integrated DEGs (including 581 upregulated and 450 downregulated genes). The top 20 upregulated and downregulated genes are shown in Fig. 5.

### Gene ontology (GO) terms for the DEGs

**Fig. 6:**
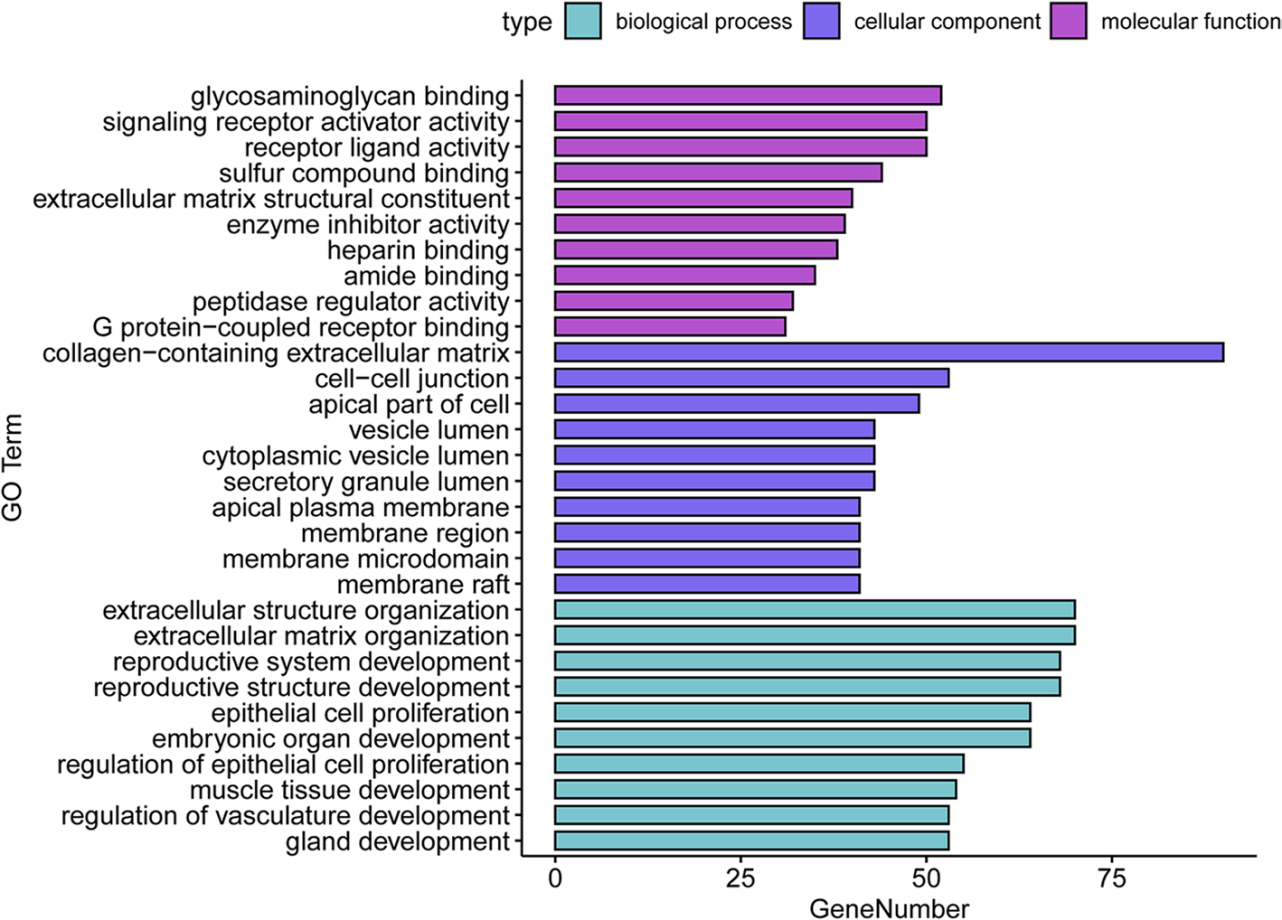
The top 10 GO terms for the DEGs in the GEO GSE7305, GSE7307, and GSE11691 datasets. Each row represents an enriched function, and the length of the bar represents the number of DEGs enriched in the corresponding function

Next, we performed GO term analysis of the DEGs in the GEO GSE7305, GSE7307, and GSE11691 datasets in endometriosis using the “clusterProfiler” package. The GO analysis data could be grouped into three categories, i.e., molecular functions, cellular components, and biological processes. Table 1 lists the top 10 GO terms for the DEGs. Using the cutoff criteria of *p* < 0.05, the three categories of GO terms are shown in Fig. 6. The molecular functions of the DEGs were mainly enriched in glycosaminoglycan binding, receptor ligand activity, and signaling receptor activator activity. The GO terms in the cellular components category were mainly involved in the collagen-containing extracellular matrix, cell–cell junction, and apical part of cells. The GO terms in the biological processes category were mainly involved in extracellular matrix organization, extracellular structure organization, and reproductive structure development.

**Table 1:** Top 10 GO terms in the DEGs from all three GEO datasets, GSE7305, GSE7307, and GSE11691

Category	Term	Count	*P*-Value
MF	glycosaminoglycan binding	52	6.33E-20
MF	receptor ligand activity	50	2.04E-06
MF	signaling receptor activator activity	50	2.73E-06
MF	sulfur compound binding	44	2.48E-12
MF	extracellular matrix structural constituent	40	1.71E-16
MF	enzyme inhibitor activity	39	3.22E-05
MF	heparin binding	38	5.89E-15
MF	amide binding	35	0.000575972
MF	peptidase regulator activity	32	2.17E-07
MF	G protein-coupled receptor binding	31	0.000100628
CC	collagen-containing extracellular matrix	90	2.28E-32
CC	cell–cell junction	53	1.12E-07
CC	apical part of cell	49	7.05E-08
CC	secretory granule lumen	43	3.63E-09
CC	cytoplasmic vesicle lumen	43	5.29E 09
CC	vesicle lumen	43	6.37E-09
CC	membrane raft	41	6.06E-08
CC	membrane microdomain	41	6.59E-08
CC	membrane region	41	1.91E-07
CC	apical plasma membrane	41	7.48E-07
BP	extracellular matrix organization	70	5.22E-21
BP	extracellular structure organization	70	6.05E-21
BP	reproductive structure development	68	5.15E-17
BP	reproductive system development	68	8.27E-17
BP	embryonic organ development	64	2.03E-14
BP	epithelial cell proliferation	64	2.51E-14
BP	regulation of epithelial cell proliferation	55	3.16E-12
BP	muscle tissue development	54	4.00E-11
BP	gland development	53	2.24E-09
BP	regulation of vasculature development	53	2.43E-09

*MF* molecular functions, *CC* cellular component, and *BP* biological process

### KEGG pathway enrichment of the DEGs

**Fig. 7:**
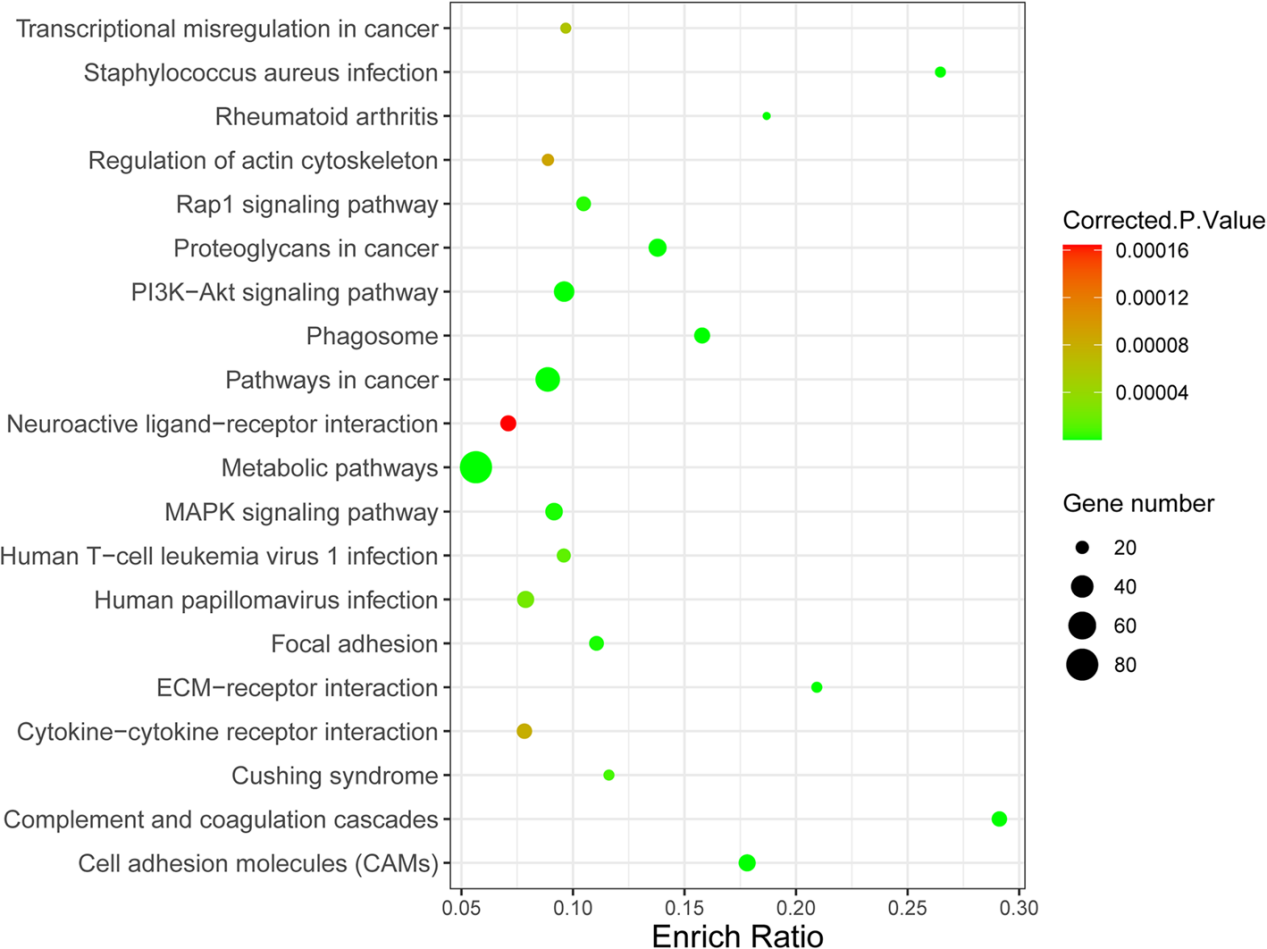
Top 20 KEGG enriched gene pathways for DEGs in the GEO GSE7305, GSE7307, and GSE11691 datasets. The horizontal axis is the ratio of the number of target proteins enriched in the pathway to the total number of proteins in the pathway, and the vertical axis represents the pathway. The size of the dot represents the number of genes enriched in the pathway. Different colors represent different correction *p* values; a color change from red to green indicates a change in the correction *p* values from large to small values and an increase in the statistical significance of enrichment

To further evaluate the DEG-related gene pathways, we performed KEGG [23–25] pathway enrichment of the DEGs in the GEO GSE7305, GSE7307, and GSE11691 datasets in endometriosis using the KOBAS software. The top 20 KEGG enriched gene pathways are shown in Fig. 7, while the top 10 KEGG enriched gene pathways are listed in Table 2. The DEGs were mostly enriched in metabolic pathways, pathways in cancer, the phosphatidylinositol 3‑kinase-protein kinase B (PI3K-Akt) signaling pathway, proteoglycans in cancer, mitogen-activated protein kinase (MAPK) signaling pathway, cell adhesion molecules (CAMs), and human papillomavirus infection. Overall, the GO term and KEGG pathway analyses suggested that immunity and inflammation were involved in the pathophysiological process of endometriosis.

**Table 2:** Top 10 KEGG enriched gene pathways for DEGs in the GEO GSE7305, GSE7307, and GSE11691 datasets

ID	Term	Count	*P* value	Genes
hsa01100	Metabolic pathways	81	2.06E-10	*CYP2J2|AOX1|AOC3|VDR|STAR|HPSE2|INMT|ACP5|UGT8|LTC4S|GGT5|GPAT3|NAMPT|PLA2G2A|P4HA3|IDO1|PAPSS2|RRM2|PDE2A|ST6GALNAC5|ALDH1A2|PAPSS1|PSAT1|CYP11A1|GCNT3|DPYD|PDE1A|GCLC|DPYS|KMO|PLA2G5|ST6GALNAC1|GLA|ASRGL1|HSD11B2|HSD17B*6|HSD1*1B1|PLPP1|NPR1|GSTZ1|PLPP2|B3GALT2|ST3GAL4|PLCB1|ENPP3|PTGIS|CA12|SORD|ALDH3B2|ENO2|GALNT15|PTGS2|HMGCR|NDUFA4L2|GATM|HGD|DSE|HSD17B2|CNDP2|CSGALNACT1|NPL|CA8|PLD1|UGT2B28|PIP5K1B|CFD|GCNT2|HMOX1|ACSL5|CYP27A1|TYMS|GPX3|NNMT|BST1|ADH1B|HSD3B2|ATP6V1C2|ASL|CHIT1|MAN1C1|CYP26A1*
hsa05200	Pathways In cancer	47	2.55E-12	*IL7R|RASGRP3|PMAIP1|PTGS2|LAMC2|FZD10|SPI1|FZD4|FZD5|FZD7|MECOM|HEY2|PAX8|JAK3|CDH1|DAPK1|WNT2B|TGFBR2|IGF1|LAMA4|IL4R|FOS|CKS2|PLCB1|PPA*RG*|FGF7|LAMC3|LEF1|CXCL12|FGFR2|FGFR3|CTNNA2|RPS6KA5|EPAS1|PLD1|FN1|ESR1|CXCL8|HMOX1|IL6|MET|RAD51|WNT2|LPAR3|AGTR1|PTCH1|LPAR4*
hsa04151	PI3K-Akt signaling pathway	34	4.10E-10	*IL7R|NGF|GHR|ITGA7|LAMC2|IGF1|LAMA4|THBS4|THBS2|NTRK2|THBS1|JAK3|COMP|ANGPT1|ERBB3|NTF3|PDGFD|IL4R|ITGB8|NR4A1|COL9A3|PPP2R2C|TNC|FGF7|LAMC3|FGFR2|FGFR3|FN1|IL6|MET|ITGA11|LPAR3|VWF|LPAR4*
hsa05205	Proteoglycans in cancer	28	8.15E-12	*HSPB2|FZD10|HPSE2|CAV2|CAV1|DCN|FZD4|FZD5|FZD7|TWIST2|ANK2|THBS1|PPP1R12B|WNT2B|ERBB3|IGF1|CTSL|GPC3|MIR10A|ITPR1|FN1|ESR1|WNT2|HOXD10|MET|IHH|ANK3|PTCH1*
hsa04010	MAPK signaling pathway	27	6.35E-08	*RASGRP3|NGF|HSPA6|TGFBR2|IGF1|MAP2K6|MAP3K8|MECOM|CACNA1D|NTRK2|DUSP4|ANGPT1|ERBB3|NTF3|PDGFD|FOS|NR4A1|FGF7|FGFR2|FGFR3|PTPN5|RPS6KA5|PTPRR|RASGRF2|MEF2C|MET|CD14*
hsa04514	Cell adhesion molecules (CAMs)	26	2.64E-13	*CLDN10|CLDN11|VCAN|CNTNAP2|NCAM1|HLA-DRA|IGSF11|CDH1|CDH3|CLDN3|CLDN4|CLDN5|CLDN7|HLA-DPB1|ITGB2|NLGN1|ITGB8|NEGR1|NFASC|VCAM1|SELE|VTCN1|PTPRC|MAG|HLA-DPA1|HLA-DQA1*
hsa05165	Human papillomavirus infection	26	1.56E-06	*PTGS2|ITGA7|ITGA11|FZD10|CCNA2|FZD5|FZD7|THBS4|THBS2|PARD6B|THBS1|COMP|WNT2B|LAMA4|ITGB8|C*O*L9A3|PPP2R2C|TNC|LAMC2|LAMC3|HEY2|FN1|WNT2|FZD4|ATP6V1C2|VWF*
hsa04145	Phagosome	24	2.06E-11	*NCF2|MRC1|HLA-DRA|C1R|THBS4|THBS2|THBS1|C3|HLA-DPB1|ITGB2|CTSL|COMP|FCGR2B|FCGR2A|ATP6V1C2|CTSS|SCARB1|HLA-DQA1|CD14|COLEC12|COLEC11|HLA-DPA1|STX18|CFD*
hsa04080	Neuroactive ligand-receptor interaction	24	1.95E-05	*PTGFR|GHR|C5AR1|PTGDR|ADCYAP1R1|P2RX7|RXFP1|S1PR1|ADRA2C|C3|EDN3|PENK|P2RY14|TRH|FPR1|CHRM3|ADM|C3AR1|GRIK2|GABRP|S1PR3|LPAR3|AGTR1|LPAR4*
hsa04610	Complement and coagulation cascades	23	8.16E-16	*VSIG4|PROS1|C5AR1|SERPINE1|SERPINA1|C4BPA|C4BPB|TFPI|C3|C7|ITGB2|CLU|THBD|CFH|F8|C3AR1|CFB|C1QB|C1QA|SERPING1|C1S|C1R|VWF*

### Protein–protein interaction (PPI) network of the DEGs

**Fig. 8:**
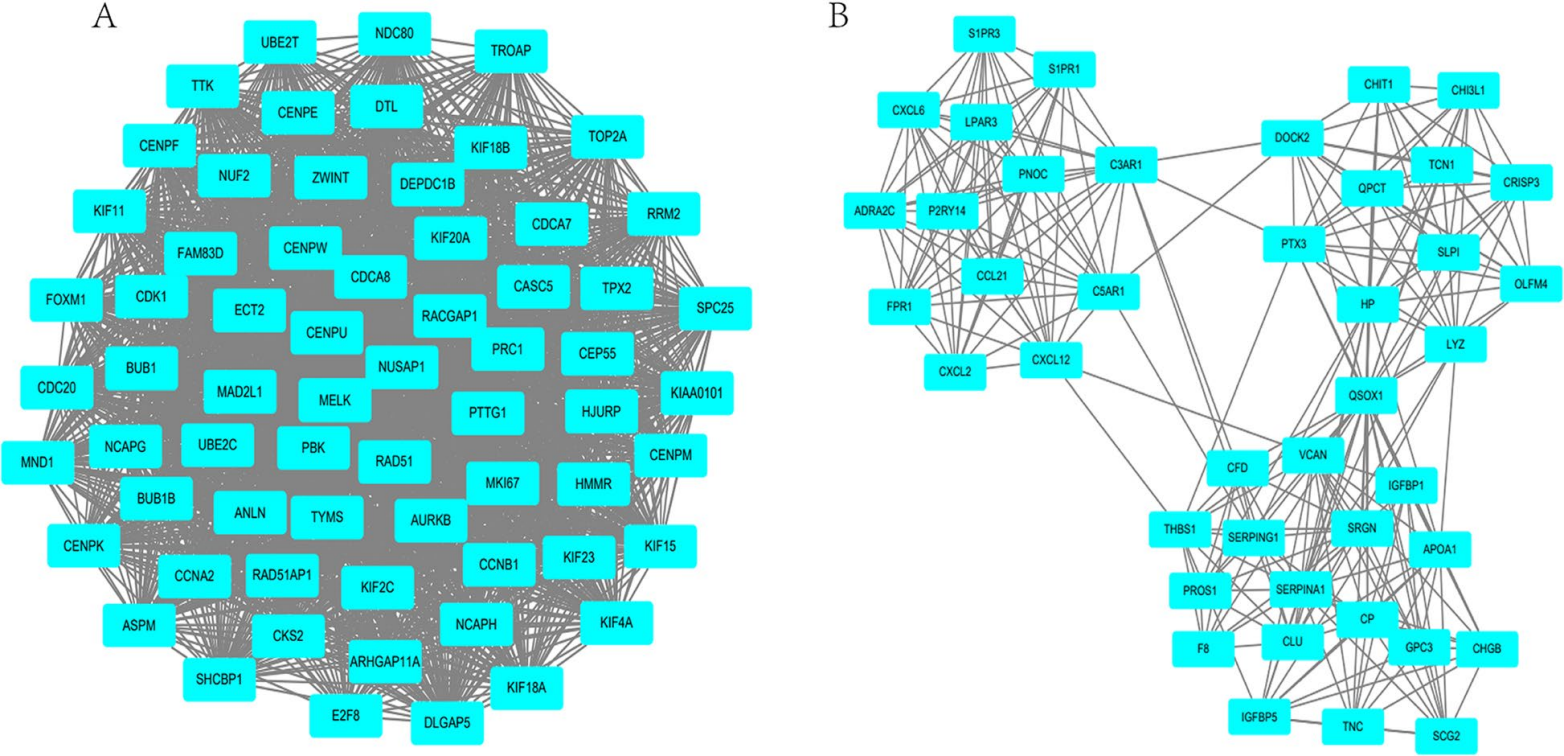
The PPI network for Module 1 (**A**) and Module 2 (**B**), which are the two most important modules filtered out from the PPI networks. The nodes represent DEGs, while the edges represent protein–protein interactions

We constructed the PPI network for the DEGs in the GEO GSE7305, GSE7307, and GSE11691 datasets using the online STRING database and analyzed the data using the Cytoscape software. Thereafter, we further screened the top 10 hub genes using the cytoHubba tool in the Cytoscape software and identified the hub genes as *IL6*, *Fibronectin 1* (*FN1)*, *CDH1*, *CXCL8*, *IGF1*, *CDK1*, *PTPRC*, *CCNB1*, *MKI67*, and *ESR1*. We also performed MCODE analysis in the Cytoscape software with the default parameters to analyze the functional modules of the PPI network. Figure 8 shows the two most important modules. The 10 hub genes were mainly involved in pathways in cancer, cellular senescence, the PI3K-Akt signaling pathway, the p53 signaling pathway, and the AGE-RAGE signaling pathway in diabetic complications. The genes in Module 1 were mainly enriched in the cell cycle and oocyte meiosis while the genes in Module 2 were mainly enriched in neuroactive ligand-receptor interactions and complement and coagulation cascades.

### Association of the hub genes with immune cells

**Fig. 9:**
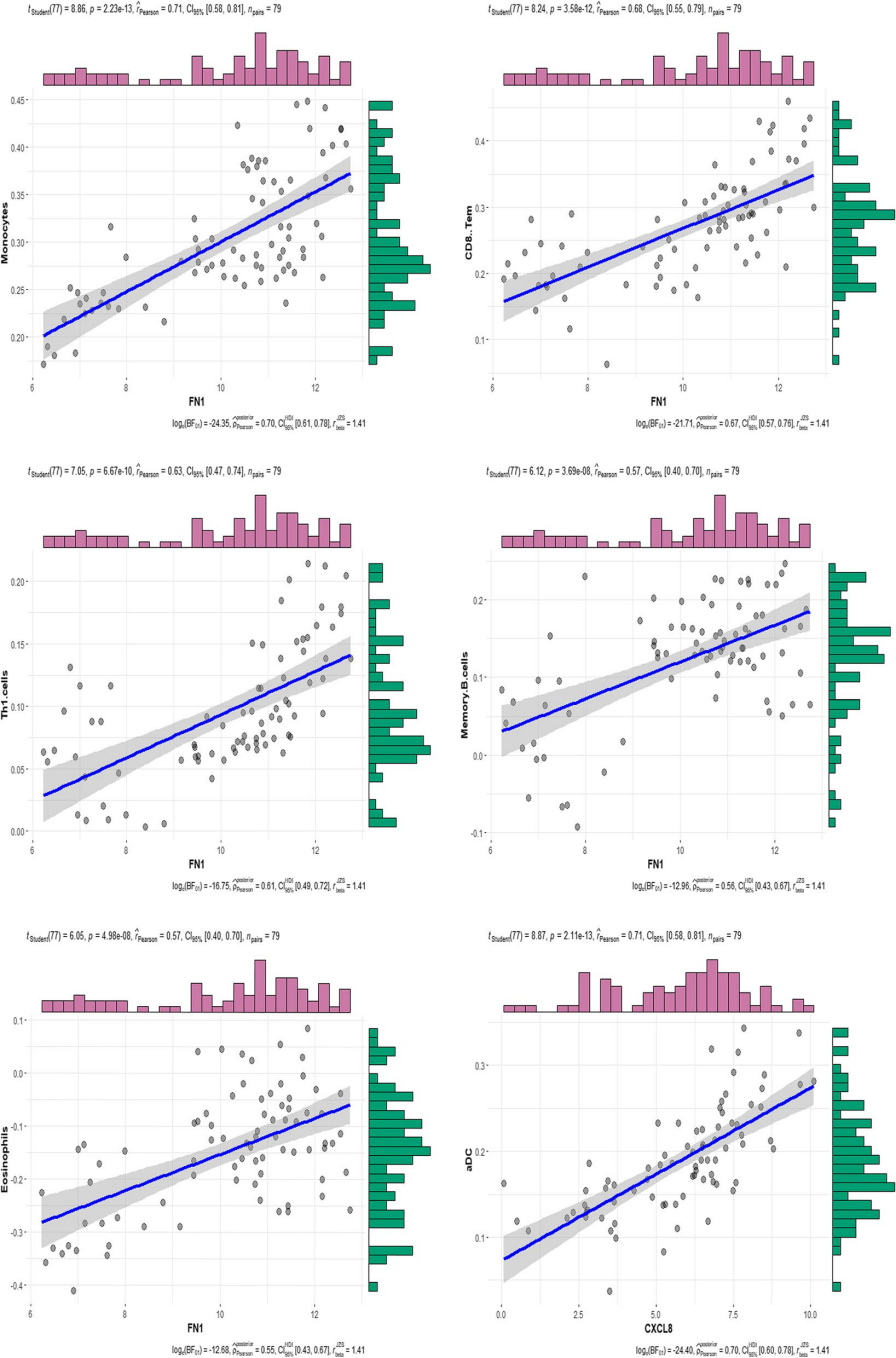
Association of the hub genes with immune cells in the GEO GSE7305, GSE7307, and GSE11691 datasets. The horizontal axis is the gene, and the vertical axis is the immune cell. The figure shows the *p* value (*p*) and the correlation coefficient (r_Pearson_).ta

Finally, we assessed the association of the 10 hub genes with the infiltration of immune cells. The expression of these 10 hub genes was associated with the scores of nine significantly different immune cell subtypes after Pearson correlation analysis (*p* < 0.05; Table 3). These 10 hub genes were significantly associated with at least three immune cells and the most significant gene was associated with eight kinds of immune cells. Th1 cells and memory B-cells were the top two cell types associated with the highest number of hub genes. The correlation index of *FN1* vs. five kinds of immune cells was greater than 0.5 and the correlation coefficient between aDCs and *CXCL8* was the highest (Fig. 9), indicating a close interplay between the immune/inflammatory response and endometriosis development and progression.

**Table 3:** Association of the hub genes with immune cells in the GEO GSE7305, GSE7307, and GSE11691 datasets

cell	HUB genes			
	IL6		FN1	
	R	*P* value	R	*P* value
CD4 + T-cells	-0.326475156	0.00332006	0.300191272	0.007189741
CD8 + T-cells	/	/	0.434110165	6.41E-05
CD8 + Tem	/	/	0.684326393	3.58E-12
Memory B-cells	-0.514066535	1.26E-06	0.571878555	3.69E-08
Eosinophils	/	/	0.567365684	4.98E-08
Monocytes	/	/	0.710500128	2.23E-13
Th1 cells	/	/	0.626348779	6.67E-10
aDC	0.695185664	1.17413E-12	/	/
pDC	/	/	0.458398123	2.15E-05
cell	CDH1		CXCL8	
	R	*P* value	R	*P* value
CD4 + T-cells	0.440221026	4.91E-05	/	/
CD8 + T-cells	0.431511911	7.17E-05	0.265114523	0.018213423
CD8 + Tem	/	/	/	/
Memory B-cells	/	/	-0.279451283	0.012626547
Eosinophils	/	/	/	/
Monocytes	/	/	/	/
Th1 cells	-0.297525423	0.007746875	0.31216276	0.00509934
aDC	/	/	0.710995393	2.11E-13
pDC	/	/	0.311325106	0.005225759
cell	IGF1		CDK1	
	R	*P* value	R	*P* value
CD4 + T-cells	0.640627142	2.04E-10	0.23983578	0.033261958
CD8 + T-cells	0.602311076	4.30E-09	/	/
CD8 + Tem	/	/	-0.430468068	7.50E-05
Memory B-cells	0.338145051	0.002304351	-0.447874672	3.49E-05
Eosinophils	0.44016858	4.92E-05	/	/
Monocytes	0.530834902	4.84E-07	/	/
Th1 cells	/	/	-0.468975666	1.30E-05
aDC	/	/	0.239747929	0.033328496
pDC	0.272858276	0.01497764	/	/
cell	PTPRC		CCNB1	
	R	*P* value	R	*P* value
CD4 + T-cells	/	/	/	/
CD8 + T-cells	0.399004506	0.000270095	/	/
CD8 + Tem	0.515246752	1.18E-06	-0.50319671	2.28E-06
Memory B-cells	/	/	-0.485778005	5.68E-06
Eosinophils	/	/	-0.294015063	0.008538147
Monocytes	0.441980272	4.54E-05	/	/
Th1 cells	0.503419828	2.26E-06	-0.548790451	1.64E-07
aDC	0.648547555	1.03E-10	/	/
pDC	0.376824412	0.00061932	/	/
cell	MKI67		ESR1	
	R	*P* value	R	*P* value
CD4 + T-cells	/	/	0.552859509	1.27E-07
CD8 + T-cells	/	/	0.403979285	0.000222414
CD8 + Tem	-0.457126222	2.29E-05	/	/
Memory B-cells	-0.57766168	2.49E-08	0.271533729	0.015493073
Eosinophils	-0.33723805	0.002371882	0.297309918	0.007793527
Monocytes	/	/	0.271319599	0.015577821
Th1 cells	-0.515808407	1.14E-06	-0.335833915	0.002479934
aDC	0.343556969	0.00193608	-0.321253615	0.003891915
pDC	/	/	/	/

R represents the Pearson correlation coefficient value; / indicates that the *p* value is greater than 0.05, which is not statistically significant

## Discussion

Our current study showed significant differences in levels of CD4^+^ and CD8^+^ T cells, CD8^+^ Tem cells, eosinophils, monocytes, Th1 cells, memory B cells, aDCs, and pDCs in endometriosis tissue samples. The key DEGs were *IL6*, *FN1*, *CDH1*, *CXCL8*, *IGF1*, *CDK1*, *PTPRC*, *CCNB1*, *MKI67*, and *ESR1*, while the 10 hub genes were associated with nine kinds of immune cells, among which *FN1* was associated with eight kinds of immune cells. The correlation of IL-8 to aDCs was the strongest, with a correlation coefficient score of 0.71. Our current study revealed that DEGs were associated with abnormal immune cell infiltration in endometriosis as well as the development of endometriosis by affecting the tissue microenvironment and the growth of ectopic endometrial cells. Poli-Neto et al. [26] also performed bioinformatical analysis and revealed differences in immune cell expression profiles among different stages of endometriosis, which were independent of the hormonal milieu; for example, they showed a high expression rate of NKT cells in endometriosis, independently of the cycle phase or disease stages, therefore, suggested a sustained stress or damage of the eutopic endometrium. Based on the analysis of immune expression profile, our current study provided the correlation between differentially expressed genes and differential immune cells as a novel strategy for further study of immune mechanism of endometriosis.

Indeed, a recent study of the GEO GSE11691, GSE23339, GSE25628 and GSE78851 datasets showed that the DEGs were closely associated with cell migration, adherens junction signaling, and hypoxia-inducible factor signaling [27]. Another recent study of the GEO GSE25628, GSE5108, and GSE7305 datasets showed that the DEGs and hub genes included genes involved in DNA strand separation, cellular proliferation, degradation of the extracellular matrix, encoding of smooth muscle myosin as a major contractile protein, exiting the proliferative cycle and entering quiescence, and growth regulation and were implicated in a wide variety of biological processes [28]. Nanda et al. [29] speculated that degradation of the extracellular matrix (ECM) in endometriosis was generally induced and the release of VEGF from the ECM promoted the angiogenesis of endometrial tissue in endometriosis patients. Thus, the combination of excessive ECM degradation and damage of cellular functions might induce the growth of ectopic endometrium and the development of endometriosis. Their pathway enrichment analysis showed the involvement of PI3K-Akt signaling, MAPK signaling, and CAMs. Honda et al. [30] reported that the PI3K-Akt and MAPK signaling pathways were activated in endometriosis. The PI3K-Akt pathway enhances cell survival, proliferation, and migration and the upregulated MAPK subfamily promotes the growth and maintenance of ectopic endometrial tissues by affecting the functions of various cytokines (such as IL-6, COX-2, and IL-8) [31]. Another study [32] revealed that specific CAMs were involved in the development of early endometriosis lesions and the unique CAM expression in endometriosis might contribute to the persistence of ectopic endometrium. In our current study, the GO terms of the DEGs were mainly enriched in extracellular matrix organization, collagen-containing extracellular matrix, and glycosaminoglycan binding, while the KEGG analysis of the DEGs were mainly enriched in PI3K-Akt signaling pathway, MAPK signaling pathway and CAMs. Our current data are consistent with the above reported research results [29–33]. However, although these studies, including our current study, were conducted using different datasets from the GEO database, the data could have identified different DEGs in endometriosis and gene pathways, indicating that further in vitro and in vivo studies are needed to confirm our data and determine the true associations or causes of endometriosis development.

Furthermore, we analyzed immune cell infiltration in endometriosis using the xCell tool and found significant differences in and high levels of CD4^+^ and CD8^+^ T cells, CD8^+^ Tem cells, eosinophils, monocytes, Th1 cells, memory B cells, aDCs, and pDCs in endometriosis vs. normal endometrial tissue samples. Endometriosis is considered a chronic inflammatory disease with known immune disorders. Growing evidence suggests that almost all subtypes of immune cells and functions are abnormal in endometriosis; for example, reduced T cell responsiveness and NK cytotoxicity, but increased B cell polyclonal activation and antibody production and peritoneal macrophages as well as changes in various inflammatory mediators and cytokines in endometriosis [33]. The ectopic endometrium contains significantly more scattered stromal CD4, CD8, and activated T cells than does the proliferative and secretory eutopic endometrium [34] and produces more cytokines, with specific immune processes to induce growth and differentiation of the ectopic endometrium. The increase of the CD4^+^/CD8^+^ T cell ratio and decrease of anti-inflammatory IL-10 could be involved in the pathogenesis of endometriosis and may secondarily affect the functions of monocytes and macrophages [35]. Immature dendritic cells (DCs) are increased in endometriosis and the surrounding peritoneum in endometriosis, but the number of mature DCs in the endometrium of patients with endometriosis is significantly lower than that in healthy endometrium, indicating that the functions of DCs in endometriosis are impaired [36]. However, in our current study, we found that level of pDC cells was increased in endometriosis. To date, only peripheral blood pDC has been studied in endometriosis samples [37] vs. the samples without endometriosis and the data showed that the number of pDC was reduced throughout the menstrual cycle. In contrast, in women with endometriosis, pDC increased as the cycle progresses, although the clinical significance of pDC dynamics throughout the menstrual cycle remains to be determined. This disorder of DC in patients with endometriosis may lead to immune escape or abnormal immune targeting of endometrial fragments that fall off during menstruation, and promote the survival of ectopic endometrium and the formation of endometriosis. Eosinophil is thought to be the most significant mammalian immune and inflammatory cells and possesses various receptors for inflammatory mediators in addition to producing a variety of pro-inflammatory and homeostatic mediators [38]. The level of CD69^+^ eosinophil occurred to be high in the peritoneal fluid of endometriosis patients, indicating that activated eosinophils accumulated in the early stages of endometriosis and played an important role in endometriosis pathogenesis [39]. Our current study further confirmed the difference in the infiltration of immune cells in endometriosis.

In addition, our current study using prospective bioinformatics analysis identified *IL6*, *FN1*, *CDH1*, *CXCL8*, *IGF1*, *CDK1*, *PTPRC*, *CCNB1*, *MKI67*, and *ESR1* as key DEGs in endometriosis. These 10 hub genes are associated with nine subtypes of immune cells in endometriosis; for example, the upregulated *FN1* expression was associated with eight subtypes of immune cells, i.e., monocytes, CD8^+^ Tem cells, Th1 cells, memory B cells and eosinophils. The correlation of aDCs with *CXCL8* was the highest, suggesting that *FN1* and *CXCL8* (IL-8) may promote the infiltration of immune cells and change the local immune microenvironment during the development of endometriosis. Efthymiou et al. [40] speculated that FN could help to shape the tumor microenvironment as the central position for the "vascular group" to not only play a key role in angiogenesis, but also enhance vascular recruitment through integrin-dependent binding of endothelial cells. FN mediates the release of inflammatory cytokines through Toll-like receptor 4 (TLR4) and the ECM to transport, mature, and activate immune cells, but prevents CD8^+^ T cells from reaching tumor cells; thereby preventing tumor cells from being destroyed by immune cells. Another study [41] showed that NKp46, the receptor on NK cells, mediated the production of IFN-γ and the latter induced *FN1* expression in tumor lesions to induce tumor metastasis. Furthermore, reduced NK cell cytotoxicity in endometriosis was not due to a decrease in their number but rather to defects in their functions [42]; therefore, there was no difference in NK cell infiltration between normal endometrium and endometriosis endometrium. However, the interaction mechanism between *FN1* and immune cells in endometriosis needs further study. *CXCL8* (IL-8), one of the first and most studied chemokines [43], acts on CXCR1 and CXCR2 receptors and is an effective neutrophil chemotactic factor to promote inflammation and angiogenesis [43]. In the current study, we found that *CXCL8* expression was higher in endometriosis than in normal endometrium. Previous studies also reported that *CXCL8* expression was significantly higher in the peritoneal fluid of endometriosis patients than that of patients without endometriosis [44, 45]. The concentration of *CXCL8* in the peritoneal fluid of patients with moderate/severe endometriosis was also higher than that of patients with mild endometriosis [46], indicating that *CXCL8* might be important in endometriosis development [47]. As a pro-inflammation chemokine, *CXCL8* participates in the development of many diseases; for example, *CXCL8* induces PD-L1 expression in macrophages to inhibit the functions of CD8^+^ T cells and promote an immunosuppressive microenvironment in gastric cancer [48]. Additionally, the expression of *CXCL8* and its receptors was found to enhance the angiogenesis, proliferation, migration, invasion, and survival of colorectal cancer cells [49] and induce the EMT and metastasis of colorectal cancer cells. Similarly, Singh et al. [50] demonstrated that a low level of *CXCL8*/IL-8 expression led to a decrease in neutrophil exudation in macular patients, suggesting that *CXCL8*/IL-8 and related signaling affected disease development. Burke et al. [51] reported that human cord blood-derived mast cells (CBMCs) produced significant amounts of *CXCL8* after the response to low levels of reovirus infection. Additionally, CBMC supernatants infected with reovirus induced substantial NK cell chemotaxis that was highly dependent on *CXCL8* and CXCR1 expression, indicating *CXCL8* played a role in the recruitment of human NK cells by mast cells. Vujanovic et al. [52] demonstrated that *CXCL8*/IL-8 was a key chemokine for DCs to recruit NK cells. *CXCL8* was reported to be involved in all processes in the development of endometriosis, including adhesion, invasion, and implantation of the ectopic tissues [53]. However, whether endometriosis depends on *CXCL8* to regulate the immune microenvironment in the development of endometriosis requires further study.

However, our current study did possess some limitations; for example, it is merely a proof of principle and further experimental investigation is needed to confirm our bioinformatics data. Moreover, this study was based on the gene expression profile provided by the Affymetrix platform to identify some important genes that can be investigated in our subsequent study to experimentally verify their roles in endometriosis development. In addition, due to the lack of detailed clinical data on GSE7307, we are unable to identify more associations between genotypes and phenotypes and to analyze the interference of the menstrual cycle stages with disease stages. Again, it is necessary to remove the batch effects to minimize such a batch effect through the PCA analysis and standardization, but the batch effect between different data sets can not be completely eliminated; in addition, although additional filters are used to eliminate samples that may be contaminated, but tissue pollution is still unavoidable. The current study, similar to previous studies [54, 55], compared very different tissues, i.e., eutopic endometrium from healthy patients, ectopic endometrium in the ovary, and ectopic endometrium in the peritoneum, which should have had very different adjacent tissues. To resolve this issue, the authors of one of the previous studies [54] first identified the probe sets that were significantly up-regulated in endometriosis compared to the control endometrium and then applied an additional set of the filters for it. This was necessary because many probe sets were the result of tissue contamination in endometriosis samples. Thus, finding a probe set that was differentially expressed between the normal ovary and normal endometrium indicated that there was a non-disease-related difference in gene expression and the probe set was removed from the endometriosis vs. the control endometrium for the differentially regulated list. Consequently, gene expression in the different normal tissues could be resolved.

## Conclusions

In the current study, we performed various bioinformatics analyses to explore the key DEGS associated with immune infiltrating cells in endometriosis. We found different levels of immune cell infiltration and a high level in endometriosis vs. normal endometrial tissues, including CD4^+^ and CD8^+^ T cells, CD8 + Tem cells, eosinophils, monocytes, Th1 cells, memory B cells, aDCs, and pDCs. The top 10 hubs were *IL6*, *FN1*, *CDH1*, *CXCL8*, *IGF1*, *CDK1*, *PTPRC*, *CCNB1*, *MKI67*, and *ESR1*. Among them, *FN1* was associated with eight subtypes of immune cells and the correlation co-efficiency between *CXCL8* and aDCs was the highest, with a value of 0.71.

## Methods

### Search and download of Gene Expression Omnibus (GEO) datasets

In this study, we first searched the GEO database (https://www.ncbi.nlm.nih.gov/geo/) using the keyword “endometriosis” and used the “GEOquery” package of the R software (version 4.0.4) to download the gene expression profiles for endometriosis (GSE7305, GSE7307, and GSE11691). The GSE7305 dataset [54] was based on the GPL570 [HG-U133_Plus_2] Affymetrix Human Genome U133 Plus 2.0 Array and included 10 samples each of ovarian endometriosis and normal endometrium. The surgical samples were taken before any medications, such as hormone therapy. GSE7305 had often been used to identify differentially expressed genes (DEGs) and analyze endometriosis in biological research related to endometriosis. A previous study showed that epithelial-mesenchymal transformation (EMT) may be induced by inflammatory cytokines and is related to smooth muscle metaplasia and fibrosis [56]. The molecular markers that regulate the development and progression of endometriosis and potential therapeutic drugs have also been identified [57]. Based on the same GSE7305 platform, GSE7307 was selected to increase the sample size for ovarian endometriosis [58], which included 18 endometriosis and 23 normal endometrial tissues. In addition, GSE11691 was added to increase the number of peritoneal endometriosis samples to enrich the data; the platform of GSE11691 [55] was the GPL96 [HG-U133A] Affymetrix Human Genome U133A Array and included 9 endometriosis tissues and 9 normal endometrial tissue samples, resulting in a total of 37 endometriosis (including 28 cases of ovarian endometriosis and 9 cases of peritoneal endometriosis as seven proliferative phase, 12 secretory phase, and 18 unknown phase samples) and 42 normal endometrial tissues (including seven proliferative phase, 12 secretory phase, and 23 unknown phase samples) in this study.

### Analyses of different immune cells in endometriosis vs. normal tissues

xCell (https://xcell.ucsf.edu/) is an online tool that can enrich gene expression in specific cell types, including 64 types of immune and stromal cells [21]. Specifically, xCell is a gene signature-based methodology that deals with thousands of pure cell types from various sources and applies a novel technique to differentiate between closely related cell types. In this study, we identified xCell signatures in three GEO datasets on endometriosis using extensive in-silico simulations and cytometric immunophenotypes. The gene signatures we selected in this study were the “Charoentong signatures (*N* = 22)” and the cut-off point was *p* < 0.05. Thereafter, we utilized the “ggplot2” package to draw split violin diagrams to visualize the differences in immune cell infiltrations in endometriosis.

### Data preprocessing and identification of differentially expressed genes

Each dataset, GSE7305, GSE7307, and GSE11691, was first normalized using the limma R package (http://www.bioconductor.org/) using the normalizing array functions. All gene expression data were then subjected to log2 transformation. Afterward, we obtained DEGs between endometriosis and normal endometrial tissues using the R package with the limma function [*p* value < 0.05 and the log fold change (FC)|> 1). The volcano map of these identified DEGs and the heatmap of the top 100 DEGs in each microarray datasets were obtained using R package.

Furthermore, we used the RRA R package to integrate the expression matrix of these three datasets and further screened the integrated DEGs (corrected *p* < 0.05, logFC > 1 or − logFC <  − 1).

### Gene ontology (GO) terms and Kyoto Encyclopedia of Genes and Genomes (KEGG) functional enrichment analysis of gene pathways

The “clusterProfiler” package was used to further explore the biological significance of the DEGs, including the GO biological process, cellular components, and molecular function terms. We next used the online KOBAS software (https://david.ncifcrf.gov/) to perform KEGG pathway enrichment analysis. A *p* value < 0.05 was used as the cutoff criterion for statistical significance.

### Construction and analysis of protein–protein interaction (PPI) network

To construct the PPI network for the DEGs in the three GEO datasets, GSE7305, GSE7307, and GSE11691, we used the Search Tool for the Retrieval of Interacting Genes Database (STRING; https://www.string-db.org/) to explore the relationship among the DEGs. Afterward, we utilized the Cytoscape software to convert the resulting data visually and screen for hub genes according to the degree of connectivity. In addition, we analyzed the functional modules in the PPI network using the Molecular Complex Detection (MCODE) plug-in in the Cytoscape software with the default parameters.

### Association of the hub genes with infiltrating immune cells

The Pearson correlation test was employed to analyze the hub genes in the infiltrating immune cells in endometriosis using the R package and the resulting data were visualized using the "ggplot" package.

### Statistical analysis

The data were summarized as mean ± SD and individual GSE7305, GSE7307, and GSE11691 data on endometriosis were analyzed for DEGs using the cut-off values of *p* < 0.05 and log fold change (FC)|> 1. The integrated DEGs of the three datasets were obtained by rank sum analysis with a corrected *p* < 0.05 and log FC > 1 or –log FC < -1 and Pearson’s rank test was used to analyze the correlation between key genes and immune cells. All statistical analyses were executed using the statistical programming language R for windows and a *p* value < 0.05 was considered statistically significant.

## Data Availability

Data is available at NCBI GEO, accession numbers: GSE7305: https://www.ncbi.nlm.nih.gov/geo/query/acc.cgi?acc=GSE7305. GSE7307: https://www.ncbi.nlm.nih.gov/geo/query/acc.cgi?acc=GSE7307. GSE11691: https://www.ncbi.nlm.nih.gov/geo/query/acc.cgi?acc=GSE11691.

## Abbreviations

GEO: Gene Expression Omnibus
DEGs: Differentially Expressed Genes
GO: Gene Ontology
KEGG: Kyoto Encyclopedia of Genes
PI3K-Akt: Phosphatidylinositol 3-Kinase-Protein Kinase B
MAPK: Mitogen-Activated Protein Kinase
CAMs: Cell Adhesion Molecules
FN1: Fibronectin 1
DCs: Dendritic Cells
aDCs: Activated Dendritic Cells
pDCs: Plasmacytoid Dendritic Cells
TNF-α: Tumor Necrosis Factor-α
VEGFR: Vascular Endothelial Growth Factor Receptor
PDGF: Platelet-Derived Growth Factor
FGF: Fibroblast Growth Factor
MMPs: Matrix Metalloproteinases
STAT4: Signal Transducer and Activator of Transcription 4
STING: Stimulator of Interferon Genes
PPI: Protein-Protein Interaction
RRA: Robust Rank Aggregation
MCODE: Molecular Complex Detection
ECM: Extracellular Matrix
TLR4: Toll-Like Receptor 4
EMT: Epithelial-Mesenchymal Transformation
CBMCs: Cord Blood-Derived Mast Cells
